# Postoperative Improvement of Visual Function Following Amplitude Increase in Intraoperative Off-Response Visual Evoked Potential (VEP) Monitoring During a Skull Base Meningioma Surgery

**DOI:** 10.7759/cureus.82563

**Published:** 2025-04-19

**Authors:** Ming X Foo, Ridzky F Hardian, Kohei Kanaya, Daishiro Abe, Satoshi Kitamura, Yutaro Sato, Tomoya Shigehara, Tetsuyoshi Horiuchi

**Affiliations:** 1 Faculty of Medicine, University of Malaya, Kuala Lumpur, MYS; 2 Department of Physiology, Faculty of Medicine, Diponegoro University, Semarang, IDN; 3 Department of Neurosurgery, Shinshu University School of Medicine, Matsumoto, JPN

**Keywords:** improvement, intraoperative monitoring, off-response, visual evoked potential (vep), visual function

## Abstract

Intraoperative visual evoked potential (VEP) monitoring does not generally predict improvement of postoperative visual function when there is an increase in the amplitude compared to the baseline recording. However, with a novel VEP monitoring method called “off-response” VEP, postoperative improvement of visual function was documented following an increase in the VEP amplitude during a skull base meningioma surgery.

The authors present a case of a patient who was diagnosed with a skull base meningioma and underwent a left frontotemporal craniotomy. The patient initially presented with a decreased visual acuity in the right eye. The best-corrected visual acuity in the right eye was 0.1 on the Landolt C chart, approximately equivalent to 20/200 on the Snellen visual acuity chart. Both off-response and conventional VEP monitoring were performed on the right eye during the surgery because the left eye was already blind. Following tumor resection, the off-response VEP recording in the right eye showed a 40% increase in amplitude, while the conventional VEP remained unchanged. The patient’s visual acuity in the right eye significantly improved after surgery.

We report a case of postoperative improvement of visual function preceded by an amplitude increase in intraoperative off-response VEP, despite unchanged conventional VEP recording during a skull base meningioma surgery. Off-response VEP is effective in monitoring visual function intraoperatively and may be highly sensitive compared to the conventional flash VEP.

## Introduction

Visual evoked potential (VEP) is the expression of the electrical activity of the visual pathways up to the optic nerve to the calcarine cortex [1]. Flash stimulation-induced VEPs, which are commonly used, are typically evaluated by measuring the peak-to-peak amplitude between the negative wave around 75 ms (N75) and the positive wave around 100 ms (P100) [2]. The P100 component is recognized as the electrical correlate of primary visual cortex activity [1].

Intraoperative VEP monitoring is commonly used to guide a surgeon’s decision-making process during neurosurgical procedures, which are done in close proximity to the visual pathway [3]. This is done to preserve visual function and avoid any unnecessary or irreversible damage to the visual nerves. VEP monitoring has been shown to predict any visual changes intraoperatively in real time [4] and allows surgeons to evaluate the aggressiveness of their approach or modify their strategies to avoid damaging the visual pathway [3].

However, flash VEP monitoring has a few shortcomings. Under the influence of general anesthesia, the VEPs obtained tend to be unstable and lack reproducibility. There are also various factors, including the suboptimal light delivery sources or the differences in effect that general anesthesia has on different individuals, which might result in inaccurate VEP readings [5]. Hence, there are conflicting reports on the association between intraoperative changes in the VEP readings and postoperative visual outcomes [6]. One of the proposed ways to address the concerns about the instability of VEP readings is to adjust the duration of light emission [7]. The flash response VEP is made up of two potentials: the “on-response” when light is emitted and the “off-response” when light emission is ceased [7]. By increasing the duration of light simulation, these two potentials would be separated and would allow the off-response to be monitored. Some studies suggest that the off-response VEP is shown to produce a more stable waveform compared to the flash VEP [8]. However, its effectiveness and underlying neurophysiology in intraoperative monitoring remain unclear.

Intraoperative VEP monitoring also poses a limitation, where it has usually been shown to predict postoperative deterioration and not postoperative visual function improvements. Some studies have shown that there are patients who showed improvement in postoperative visual function despite not showing any improvements during the intraoperative monitoring [9,10]. There is also a lack of substantial studies on the threshold percentage of the amplitude increase that a VEP reading has to achieve to be considered a significant finding.

To illustrate this hypothesis, we discuss a case where a patient showed an improvement in off-response VEP during intraoperative monitoring, which resulted in an improved visual function after the surgery despite the flash VEP showing a stable response.

## Case presentation

Patient history and examination

**Figure 1:**
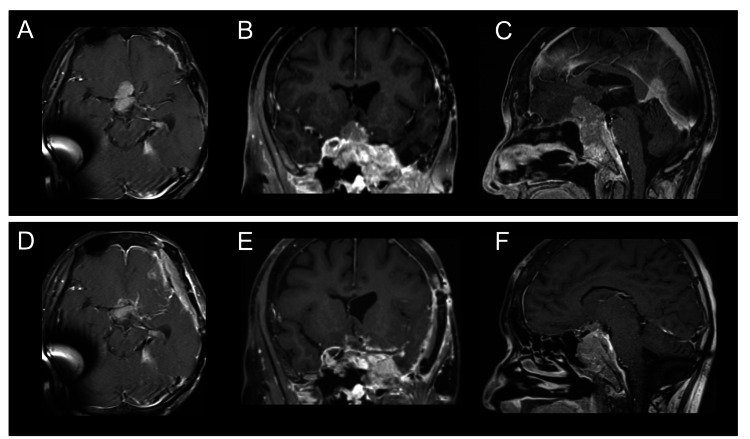
Pre and postoperative contrast-enhanced T1-weighted images Preoperative axial (A), coronal (B), and sagittal (C) sections of contrast-enhanced T1-weighted images revealed a skull base tumor extending to the suprasellar region. Postoperative axial (D), coronal (E), and sagittal (F) sections of contrast-enhanced T1-weighted images showed partial removal of the tumor around the optic nerves.

A woman in her 50s presented to our institute with a complaint of a decrease in visual acuity in the right eye. The best-corrected visual acuity in the right eye was 0.1 on the Landolt C chart, approximately equivalent to 20/200 on the Snellen visual acuity chart. Her left eye was blind, while her right eye showed temporal hemianopia on Goldmann perimetry. There was no impairment of the eye movement in her right eye, but limited movement and ptosis were present in her left eye. There was also hypoesthesia and numbness in the regions innervated by the left maxillary (V2) nerve. Magnetic resonance imaging (MRI) revealed that the skull base tumor extended to the suprasellar region, which caused the deterioration of her right visual function (Figures 1A-1C). 

She had a 20-year history of recurrent meningiomas and multiple surgeries. She had lost visual function of her left eye. The suprasellar lesion rapidly enlarged, and her visual function deteriorated despite the previous surgical treatment via endoscopic endonasal approach. Therefore, a left frontotemporal craniotomy was decided with an aim to preserve her visual function. The procedure was performed under VEP, electroretinography (ERG), and motor evoked potential (MEP) to prevent any further neurological impairment.

Operation and postoperative course

Both on-response VEP (flash VEP) and off-response VEP, ERG, and MEP readings were recorded using the Neuropack® Sigma evoked potential measuring system (Nihon Kohden, Japan). Goggles with red light-emitting diodes (Unique Medical Co. Ltd., Japan) were secured in place over the patient’s closed eyelid on the right eye, and aluminum foil was placed above the goggles to prevent light leakage. VEP for the left eye was not monitored because the left eye was already blind. Mean values of both on-response VEP and off-response VEP readings were obtained after stimulation of the right eye. The average stimulation time for the on-response VEP was one flash per 40 msec, while the off-response VEP was one flash per 400 msec; an average of 100 flashes were recorded to obtain the mean value for both VEP readings. VEP recordings were performed in a three-channel montage, with electrodes placed on the bilateral earlobes (A1, A2), the left occiput (O1), the occipital midline (Oz), and the right occiput (O2), while the ERG recordings were recorded via electrodes placed on the bilateral canthi (X1, X2). Initial on-response VEP and off-response VEP could be recorded with a stimulation intensity of 15mA. MEP was performed via transcranial stimulation with corkscrew electrodes on cervical vertebrae (C3, C4), and recordings were obtained via the bilateral abductor pollicis brevis (APB) and extensor muscles of the forearm.

**Figure 2:**
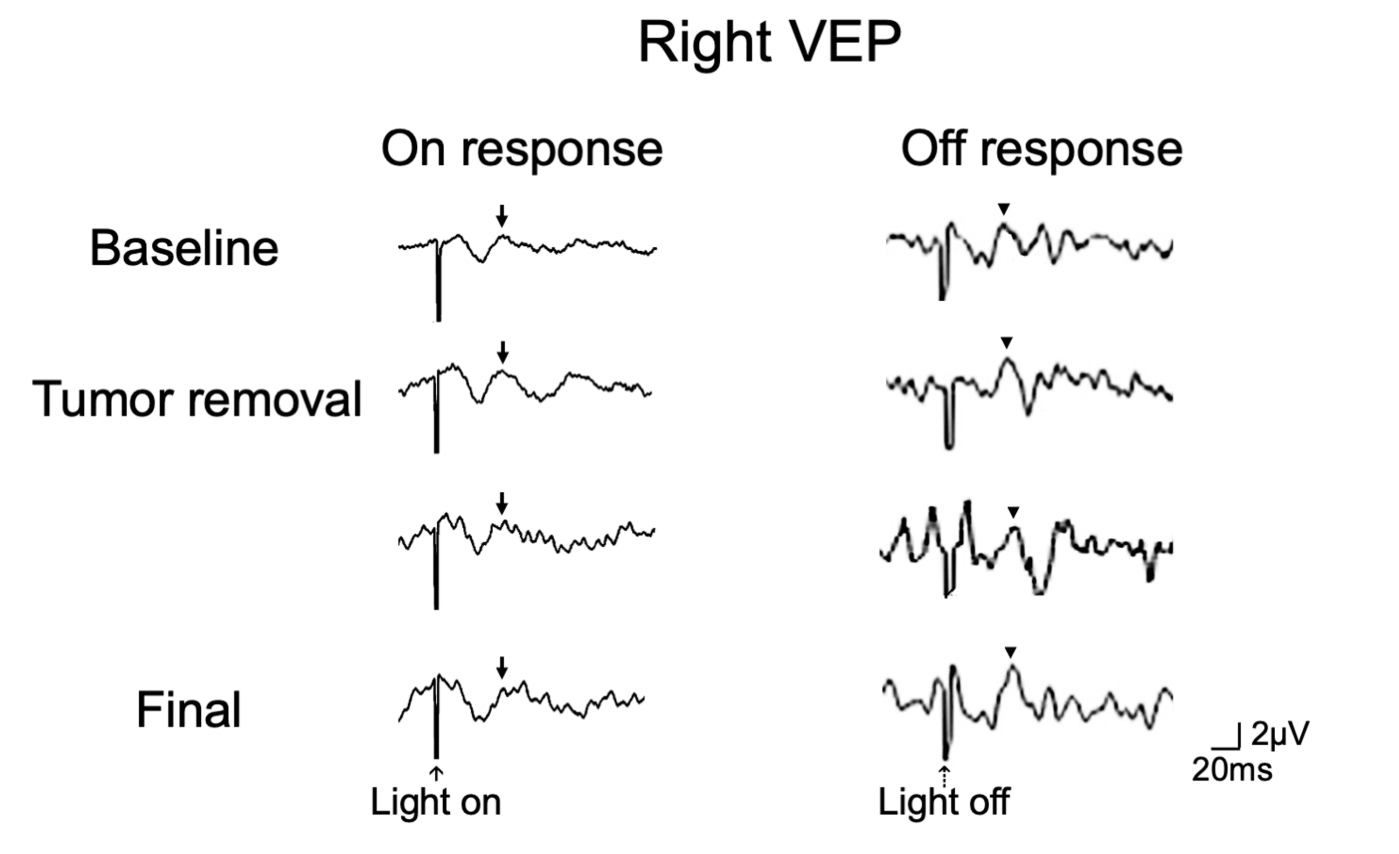
Waterfall display of intraoperative right VEP monitoring with on-response (left) and off-response (right) monitoring from the start of the surgery (baseline recording) until after the tumor removal. Off-response VEP monitoring (arrowheads) detected a 40% improvement in amplitude (from 2.8 μV to 4.0 μV) with latencies of approximately 70 ms during the tumor removal procedure around the right optic nerve, while the on-response VEP (flash VEP) monitoring (arrows) was unchanged throughout the procedure. VEP: Visual evoked potential

General intravenous anesthesia was induced and maintained throughout the surgery. A left fronto-temporal craniotomy was performed with the left sylvian fissure approach. Once the craniotomy was performed, the dura mater was incised, and adhesions to the brain surface were observed. The left sylvian fissure was opened, and the left internal carotid artery, optic nerve, and the tumor were identified. The tumor was elastic, hard, and adhered to the surrounding tissues. The tumor was removed via the prechiasmatic cistern; an effort was made to identify the right internal carotid artery and optic nerve. The right optic nerve was identified, and the surrounding tumor was dissected and excised. The baseline amplitudes of the on-response and off-response VEPs were 1.6 μV and 2.8 μV, respectively, with latencies of approximately 70 ms. During resection of the tumor near the right optic nerve, the amplitude of the off-response VEP increased by approximately 40% to 4.0 μV, while the on-response VEP (flash VEP) remained unchanged (Figure 2). The final flash VEP and off-response VEP could be recorded with a stimulation intensity of 15mA. There was no meaningful change to the flash VEP during the surgery. However, the off-response VEP demonstrated a reproducible improvement in amplitude following tumor removal around the right optic nerve, and this improvement persisted through the end of the surgery. The MEP reading was stable throughout the surgery as well, and no changes were seen in the motor function postoperatively.

Postoperative MRI showed partial removal of the tumor (Figures 1D-1F), and the histopathological diagnosis was meningothelial meningioma (World Health Organization (WHO) Grade 1). Postoperative recovery was uneventful without any complications. A remarkable improvement in the right visual function was observed just after the surgery, with an increase of the best-corrected visual acuity to 0.5 on the Landolt C chart, approximately equivalent to 20/40 on the Snellen visual acuity chart. There was no change in the right visual field on Goldmann perimetry postoperatively. The patient was then discharged with follow-ups to the neurosurgery and ophthalmology clinics. Repeat MRI during the six-month follow-up showed no recurrence of the tumor.

## Discussion

Due to its instability and lack of reproducibility, flash VEP monitoring has received conflicting studies regarding its usefulness as a means for monitoring and predicting a patient’s postoperative outcomes [11,12]. Even so, many studies have concluded that in an ideal setting, flash VEP monitoring is only useful for detecting the deterioration of the visual function and is often unable to predict the improvement of function postoperatively [13]. As such, many alternatives have been suggested to address the issue of flash VEP monitoring and propose a solution to the lack of stability and reproducibility.

One such method is to prolong the emission time so that the off-response VEP, formed from the absence of light emission, would be separated from the on-response VEP, the potential when light is emitted. This provides the possibility of recording and analyzing the off-response VEP [8]. In flash VEP monitoring, both the on-response and off-response potentials are combined and would produce inaccurate results. One of the reasons is that both off-response and on-response show opposite changes in emission intensity. The on-response would show a reduction in variation of peak latency when the emission intensity is high, while the off-response would show the opposite, that is, a reduction of variation in peak latency when the emission intensity is lower. As such, both on-response and off-response components contain distinct stabilizing elements, which may contribute to the instability of conventional flash VEP recordings [7]. In contrast, off-response VEP allows for separate analysis of the "on" and "off" components, enabling more reliable interpretation of each potential. Another theory suggests that the off-response provides a more stable reading because, in the presence of multiple obstacles obstructing light from reaching the retina, the reduction in light quantity results in a more consistent potential being recorded [7].

This case is a rare instance where the off-response VEP was improved during operation and resulted in an improved visual function despite the stable and constant flash VEP. Studies have suggested that a decrease in visual function and VEP readings can be attributed to two main causes, namely the contraction and retraction of the nerve fibers or ischemic changes due to bleeding [14]. In this case, the off-response VEP improvement can be seen after the resection of the tumor around the optic chiasma, which would explain the postoperative visual function improvement. As previously mentioned, intraoperative VEP monitoring rarely predicts postoperative visual improvements. It is also worth noting that only the off-response VEP showed improvement intraoperatively, while the flash VEP remained relatively stable throughout the surgery. A few hypotheses may explain this occurrence. Due to its stability, the off-response VEP can more accurately detect the increase in VEP after the decompression of the optic nerve following tumor removal. The flash VEP, which consists of both "on" and "off" responses, may have cancelled each other out due to the difference when reacting to changes in light intensity and stability factors. 

This case report aimed to illustrate the possibility of the off-response VEP to be used as a more stable alternative alongside the flash VEP. However, it should be noted that this might be a singular instance, and more data needs to be analyzed before knowing if the off-response VEP monitoring is able to predict postoperative visual improvements. 

It should also be noted that several limitations exist before we can determine the accuracy of off-response VEP and whether VEP may be used as a predictor for visual improvement. Firstly, this is only a single case report, and as such, more reports on similar occurrences and results are needed to have substantial proof of our hypothesis. We also do not know the minimum threshold for improvement during the intraoperative VEP monitoring for it to be significant for a postoperative visual function improvement. At the time being, studies have shown that postoperative visual function improvement can occur even without an improvement in intraoperative VEP reading [9,10]. As such, more research should be done on this, as it might pave the way for having not only a potential alternative for the unstable conventional flash VEP but also a reference point for a predictor of intraoperative visual improvement, which would further guide surgical decision-making.

## Conclusions

We report a case of postoperative improvement of visual function following amplitude increase in intraoperative off-response VEP, despite unchanged conventional flash VEP recording during a skull base meningioma surgery. Off-response VEP is effective in monitoring visual function intraoperatively and can be used as an alternative to the conventional flash VEP.
